# Association between frailty status and pain, balance, and quality of life in patients with lumbar spinal stenosis

**DOI:** 10.55730/1300-0144.6158

**Published:** 2026-01-10

**Authors:** Musa GÜNEŞ, Aydın Sinan APAYDIN

**Affiliations:** 1 Department of Physiotherapy and Rehabilitation, Faculty of Health Sciences, Karabük University, Karabük, Turkiye; 2 Department of Neurosurgery, Faculty of Medicine, Karabük University, Karabük, Turkiye

**Keywords:** Lumbar spinal stenosis, frailty, pain, balance, disability, quality of life

## Abstract

**Background/aim:**

Frailty is a clinical syndrome that affects individuals physically and psychosocially. However, the association between lumbar spinal stenosis (LSS) and frailty remains unclear. This study aimed to compare pain, balance, disability, fear of falling, and quality of life between patients with LSS with and without frailty.

**Materials and methods:**

This cross-sectional study included 43 frail and 48 nonfrail patients with LSS according to the frailty criteria of Fried et al. Pain intensity (numeric rating scale [NRS]), static balance (single leg stance test [SLST]), dynamic balance (Timed Up and Go test [TUG]), disability (Oswestry Disability Index [ODI]), fear of falling (Falls Efficacy Scale–International [FES-I]), and quality of life (Short form-36 [SF-36]) were assessed.

**Results:**

Frailty was observed in 47.3% of patients, and baseline characteristics were similar between the groups (p > 0.05). NRS scores for low back and leg pain, ODI and FES-I scores, and TUG test durations were significantly higher in frail patients with LSS than in nonfrail patients (p < 0.05). SLST durations and SF-36 subscale scores were significantly lower in frail patients with LSS (p < 0.05). TUG duration (p = 0.001, OR = 1.482), physical activity level (p = 0.007, OR = 0.999), and the SF-36 vitality subscale (p = 0.031, OR = 0.968) were significantly associated with frailty in the logistic regression model (p < 0.001).

**Conclusion:**

Frail patients with LSS experience greater pain intensity, balance disorders, disability, fear of falling, and poorer quality of life. Moreover, reduced mobility, lower physical activity levels, and decreased vitality are associated with a higher likelihood of frailty in patients with LSS. Therefore, early assessment of frailty in patients with LSS and the implementation of personalized rehabilitation approaches may help mitigate its negative impact.

## 1. Introduction

Lumbar spinal stenosis (LSS) is a degenerative spinal condition characterized by nerve root compression resulting from progressive narrowing of the spinal canal or neural foramen. Clinically, it presents with chronic symptoms such as low back pain, neurogenic claudication, numbness in the lower extremities, muscle weakness, and a marked reduction in walking capacity [1,2]. The prevalence of LSS increases with advancing age and is more common in individuals older than 60 years [3]. In addition to age-related chronic conditions, LSS is associated with loss of muscle strength, physical inactivity, impaired balance, and functional impairments [1,4,5]. Consequently, quality of life decreases, while the likelihood of disability and falls increases [6–8]. Muscle weakness and reduced physical functioning are potential predictors of frailty [7,9].

Frailty is a clinical syndrome characterized by multisystem involvement, leading to diminished functional reserve and reduced resilience to physiological stressors. Loss of strength, decreased physical activity, unintentional weight loss, reduced mobility, and exhaustion are components of frailty [9]. Frailty progresses more rapidly with ageing and is associated with an increased risk of balance impairment, disability, and mortality [10,11]. Additionally, frailty is associated with increased fear of falling, which negatively affects physical and psychosocial health [12]. Therefore, frailty, particularly in the presence of chronic diseases, can profoundly affect individuals’ quality of life and long-term health outcomes [13].

Previous studies in individuals with low back pain (LBP) have confirmed a bidirectional relationship between frailty and LBP [14,15]. According to one study, greater frailty in older adults with acute LBP was associated with increased disability and impairment in the physical component of quality of life [15]. However, the relationship between LSS—which causes lower extremity pain in addition to low back pain—and frailty has not been sufficiently investigated. Considering the decline in physical function associated with frailty, LSS and frailty may be related due to overlapping clinical symptoms [7]. The prevalence of frailty in patients with LSS was reported as 24.8% and 41.5% [7,16]. Additionally, only one study has shown that symptom severity and disability in LSS are associated with frailty [7].

In the ageing population, LSS and frailty may adversely affect patients, leading to poorer health outcomes and increased mortality [1,11]. In this context, the presence of frailty in patients with LSS may be associated with worsening of existing symptoms. Such worsening has been shown to be associated with increased disability [7]. However, the relationship between balance, fear of falling, and quality of life in patients with LSS and frailty remains unclear. Understanding the relationship between these factors and frailty may be important for maintaining physical function and optimizing the clinical management of LSS. Therefore, this study aimed to compare pain, balance, disability, fear of falling, and quality of life between frail and nonfrail patients with LSS.

## 2. Materials and methods

### 2.1. Participants

This cross-sectional study was conducted among 91 patients with LSS who were followed in the neurosurgery department between May and July 2025. The study included patients who were radiologically diagnosed with LSS as a stenotic lesion of the lumbar spine on magnetic resonance imaging by a neurosurgeon, who clinically presented with neurogenic intermittent claudication and one or more related symptoms (pain or numbness), were aged between 45 and 80 years, and were able to walk without support or assistive devices. Exclusion criteria were as follows: a history of lumbar spinal surgery; spinal infections or tumors; lower extremity joint diseases; spinal fractures; chronic diseases affecting balance or gait; visual or vestibular system disorders; and cerebrovascular or neuromuscular diseases. The study was approved by the university ethics committee (2025/2280) and was conducted in accordance with the Declaration of Helsinki. The hospital where the study was conducted provided institutional approval (approval no: E-34771223-774.99-275790270). All patients provided written informed consent prior to participation in the study (clinical trial no: NCT06959355). Patients with LSS included in the study were categorized into frail and nonfrail groups according to frailty status.

### 2.2. Assessments

#### 2.2.1. Frailty assessment

Frailty was evaluated according to the five-component criteria established by Fried et al. [9]. Frailty was defined as the presence of three or more of the following criteria: weight loss, muscle weakness, decreased mobility, exhaustion, and physical inactivity [9].

Weight loss was defined as unintentional loss of more than 4.54 kg during the previous year [9]. Handgrip strength was assessed using a Jamar hand dynamometer (Fabrication Enterprises Inc., Irvington, NY, USA) to determine muscle weakness. Participants were seated with back support, and the test was performed three times on the dominant side with the elbow flexed at 90°. The highest value was recorded. Cut-off points for handgrip strength were based on the values recommended by Fried et al. [9] and were adjusted for sex and body mass index (BMI). Exhaustion was assessed using two items from the Center for Epidemiologic Studies Depression (CES-D) scale: “How often in the last week did you feel that everything you did was an effort?” and “How often in the last week did you feel that you could not get going?”. Scoring ranged from 0 to 3: 0 indicated rarely or never (<1 day), 1 indicated sometimes (1–2 days), 2 indicated moderately (3–4 days), and 3 indicated most of the time. Participants who scored 2 or 3 on either item were classified as exhausted [7,9,17]. Mobility was assessed using a 4.57 m walking test, with the start and end points marked by cones. Participants were instructed to walk at their usual pace, and the time was recorded. Slowness was adjusted according to sex and height [9]. Participants’ physical activity levels during the previous week were assessed using the international physical activity questionnaire (IPAQ) [18,19]. According to frailty criteria, low physical activity was defined as less than 150 min per week of moderate-intensity activity or 75 min per week of vigorous-intensity activity [7,9].

#### 2.2.2. Pain severity

Low back and leg pain severity was evaluated using the numeric rating scale (NRS). The scale is scored from 0 to 10 (0–10), with 10 indicating the worst imaginable pain, and participants were asked to indicate the severity of pain they experienced [5].

#### 2.2.3. Balance

Static balance was assessed using the eyes-open single leg stance test (SLST). This test is valid and can be applied quickly and easily in clinical settings. Participants were instructed to stand on the affected leg and raise the contralateral knee to a 90° angle. The test was repeated three times, and the best result was recorded. In cases of bilateral involvement, both legs were tested, and the best time for each extremity was recorded; the average of these values was then used [20].

Mobility and dynamic balance were evaluated using the Timed Up and Go (TUG) test. In the TUG test, participants were instructed to stand up from a chair, walk 3 m at their usual pace, turn around, return to the chair, and sit down, and the time taken was recorded [21].

#### 2.2.4. Disability

Functional disability related to LBP in activities of daily living was assessed using the Oswestry Disability Index (ODI). The ODI consists of 10 items (e.g., walking, standing, sitting, personal care, sleeping, social life, sex life, traveling, working, and lifting), each rated from 0 to 5. Total scores range from 0 to 100. Higher scores indicate greater levels of disability [22].

#### 2.2.5. Fear of falling

The Falls Efficacy Scale–International (FES-I) was used to assess fear of falling in activities of daily living. The FES-I consists of 16 items, each scored from 1 to 4. Total scores range from 16 (no concern) to 64 (extreme concern) [23].

#### 2.2.6. Quality of life

The Short Form-36 (SF-36) was used to assess health-related quality of life. Scores range from 0 to 100, with higher scores indicating better health status. The questionnaire consists of 36 items grouped into eight subscales: physical functioning, bodily pain, role limitations due to emotional problems, social functioning, general health, role limitations due to physical problems, mental health, and vitality [24,25].

### 2.3. Statistical analysis

SPSS version 25.0 (IBM Corp., Armonk, NY, USA) was used to analyze the data. Sample size was calculated using G*Power software (Heinrich Heine University Düsseldorf, Düsseldorf, Germany). Sample size calculation was based on ODI results for frail and nonfrail (robust and prefrail) groups reported in a previous study of patients with LSS [7], assuming an effect size of d = 0.66, an alpha level of 0.05, and 80% power, yielding a minimum of 30 participants per group.

Normality of the data was assessed using the Kolmogorov–Smirnov test and histogram plots. Normally distributed numerical variables were presented as mean ± standard deviation (SD) with 95% confidence intervals (CI), whereas nonnormally distributed variables were presented as median and interquartile range (IQR). Categorical variables were compared using the chi-square test. Group comparisons were performed using the independent samples t-test for normally distributed data and the Mann–Whitney U test for nonnormally distributed data. The relationships between frailty and other parameters were analyzed using Pearson or Spearman correlation analyses, as appropriate. Correlation coefficients were interpreted as follows: r > 0.89, very strong; 0.70–0.89, strong; 0.40–0.69, moderate; and 0.20–0.39, weak [26]. Patients were categorized as frail and nonfrail. Logistic regression analysis was performed using the forward stepwise method to identify variables independently associated with frailty. The level of statistical significance was set at p < 0.05.

## 3. Results

A total of 95 patients with LSS were screened, three patients were excluded due to previous surgery, and one patient declined to participate. According to the Fried frailty criteria, of the 91 patients included in the study, 43 (47.3%) were frail and 48 (52.7%) were nonfrail. The comparison of demographic and baseline characteristics between frail and nonfrail patients with LSS is presented in Table 1. No significant differences were found between the demographic and baseline characteristics of the two groups (p > 0.05), except for handgrip strength and the 4 m walking test (p < 0.001; Table 1).

The prevalence of weakness, reduced mobility, low physical activity, and exhaustion was significantly higher in the frail LSS group than in the nonfrail LSS group (p < 0.001). Because handgrip strength and the 4 m walking test are components of frailty, they were analyzed separately as frailty-related parameters (p < 0.001; Table 2). Frail patients had significantly lower handgrip strength values and longer 4 m walking test times than nonfrail patients (p < 0.001; Table 1).

Frail patients with LSS had significantly higher low back and leg pain intensity, TUG test duration, and ODI and FES-I scores than nonfrail patients (p < 0.05). Additionally, frail patients with LSS had significantly lower SLST duration and IPAQ and SF-36 subscale scores (p < 0.05; Table 3). Based on the ODI score, the post hoc study power was calculated as (1 – β) = 99.9%.

In patients with LSS, a statistically significant moderate correlation was observed between frailty level and NRS, TUG, ODI, and FES-I scores (r = 0.424–0.626; p < 0.001). A statistically significant negative correlation was found between frailty level and SLST, IPAQ, and SF-36 subscale scores (p < 0.001; Table 4).

Variables predicting frailty status were examined using logistic regression analysis. The logistic regression model was statistically significant (χ^2^(3) = 53.278; p < 0.001). The model explained 51.9% of the variance in frailty (Nagelkerke R^2^) and correctly classified 82.4% of patients. A 1 s increase in TUG test duration was associated with a 1.48-fold higher likelihood of frailty. Higher physical activity scores were associated with a lower likelihood of frailty. Additionally, a one-point increase in the SF-36 vitality subscale score was associated with a 3.2% lower likelihood of frailty (Table 5).

## 4. Discussion

This study demonstrated that patients with frail LSS experienced greater pain, balance impairment, disability, fear of falling, and poorer quality of life than nonfrail patients. In addition, higher frailty levels were associated with worsening of these parameters. In the present study, 47.3% of patients with LSS were frail, and longer TUG test duration was associated with a higher likelihood of frailty. Concurrently, higher physical activity levels and better quality of life subscale scores were associated with a lower likelihood of frailty.

Frailty is becoming increasingly common with population ageing and is associated with adverse health outcomes [11,13]. This study assessed frailty using the Fried criteria, a multicomponent assessment method [9]. In patients with LSS, all frailty components except weight loss were more prevalent in the frail group. Additionally, patients with frail LSS exhibited lower handgrip strength, slower walking speed, and lower physical activity levels. Kim et al. [7] reported poorer outcomes in patients with LSS than in healthy controls across Fried frailty components other than weight loss, consistent with our findings. In LSS, decreased handgrip strength is also associated with muscle weakness and deterioration in walking ability [4]. These findings suggest that physical inactivity may contribute to the relationship between LSS and frailty through loss of muscle strength [7]. Together, these findings may explain the presence of generalized muscle weakness in LSS and reduced functional capacity associated with frailty. Thus, evaluating frailty in patients with LSS is essential, particularly given its increasing prevalence with advancing age [3,7].

Previous studies have reported frailty prevalence rates in LSS of 41.5% and 24.8%, respectively [7,16]. The present study demonstrated a higher prevalence of frailty (47.3%). Frailty is associated with more severe symptoms and disability in patients with LSS [7,14]. Therefore, worsening symptoms in LSS may contribute to walking intolerance and physical inactivity, potentially exacerbating muscle weakness and frailty [7]. Compared with the study by Kim et al. [7], the present study demonstrated greater disability severity, higher physical inactivity rates, reduced mobility, and increased muscle weakness in patients with LSS. Worsening symptoms and functional deterioration may have contributed to the higher prevalence of frailty observed in this study. Additionally, Nagai et al. [16] assessed frailty using a different scale in relation to chronic disease risk and postoperative complications. These methodological and clinical differences may help explain the higher prevalence of frailty observed in the present study.

Ageing and LSS are associated with degenerative changes in the spine, leading to increased pressure on the nerve roots. As a result, pain radiating to the low back and legs may occur and is often chronic [27]. Previous studies have confirmed the presence of moderate to severe pain in patients with LSS and that this pain may persist for many years [6,28]. Furthermore, the severity of low back and leg pain observed in this study was higher than that reported in several previous studies [5,6,29]. Pain intensity may increase in relation to compression of the dural sac [30]. Additionally, women with LSS may be more sensitive to pain and therefore experience more severe low back and leg pain [31]. Compared with previous studies [5,6,29], the higher proportion of women, the presence of multilevel stenosis, and potentially more severe stenotic changes in the current study may have contributed to greater pain severity. These factors may contribute to pain becoming chronic and more burdensome for affected individuals. Chronic and more severe pain is associated with an increased likelihood of developing frailty [32]. Pain is associated with frailty in patients with LBP [14,15]. However, the association between frailty and pain in LSS remains unclear. The present study demonstrated that patients with frail LSS experienced greater low back and leg pain than nonfrail patients and that higher frailty levels were associated with increased pain intensity. LSS may be associated with poor balance, walking intolerance, and physical inactivity due to low back and leg pain [6,7]. This situation may contribute to muscle weakness, which in turn may worsen frailty [7]. In LSS, decreased handgrip strength is associated with increased severity of neurogenic claudication, reduced overall muscle strength and mass, and decreased walking speed [4]. In the present study, lower handgrip strength in frail patients with LLS was associated with poorer functional status, helping to explain the relationship between LSS and frailty. In addition, pain may increase individuals’ vulnerability to stressors and reduce functional performance by promoting physical inactivity [33]. Therefore, frailty may be more prevalent in patients with LSS, in whom pain represents a major limiting factor. Furthermore, the higher pain severity observed in this study may have contributed to greater reductions in functional performance, thereby influencing frailty. Taken together, these findings support a bidirectional relationship between pain and frailty in LSS.

Pain and postural changes in LSS may adversely affect balance [1,5,28]. Balance may be disrupted in LSS due to a forward-leaning posture that develops to reduce nerve root pressure [1]. Additionally, physical inactivity associated with pain in LSS may exacerbate age-related muscle weakness and atrophy [7]. Frailty is also considered a nonvestibular contributor to balance disorders, and dysfunction of postural muscles is associated with frailty [10]. Shinohara et al. [10] reported that frail older women exhibited poorer stability limits, sensory orientation, and postural control, with impaired balance as assessed by the BEST test. Because LSS and frailty may affect balance through similar mechanisms, their coexistence may increase the likelihood of balance impairment. This study demonstrated for the first time that static balance was poorer in patients with frail LSS than in those without frailty. Additionally, higher frailty levels were moderately associated with shorter SLST duration. Frailty, as a constellation of impaired physiological mechanisms affecting multiple organs and systems [9], when present alongside LSS, may exacerbate musculoskeletal impairments and further compromise balance and stability.

Previous studies in LSS have also shown that dynamic balance and mobility are impaired [5,6,21]. Frailty is also associated with slowness [9], and in the present study, patients with frail LSS demonstrated poorer performance on the walking test, which represents one of the frailty components. The TUG test is frequently used to evaluate functional mobility required for activities of daily living [21]. In this study, TUG test duration was longer in patients with frail LSS than in nonfrail patients. Additionally, a 1 s increase in TUG test duration was associated with a 1.48-fold higher likelihood of frailty. In this context, TUG test findings reflect reduced mobility, which is a key component of frailty. Pain and nerve dysfunction in LSS may contribute to physical inactivity, reduced mobility, and fatigue [7]. The coexistence of frailty may further increase the severity of these existing problems. Therefore, frailty may adversely affect activities of daily living by further decreasing mobility in LSS. Frailty in patients with LSS should be assessed early to reduce dependency in activities of daily living and improve functional outcomes.

Previous studies have demonstrated an association between LBP and frailty and have emphasized that frailty is associated with increased disability in individuals with LBP [15,34]. LSS, which is associated with low back and leg pain, is linked not only to increased disability [6,28] but also to frailty, which itself is associated with the development and progression of disability [35]. Only one study in the literature, by Kim et al. [7], has reported that frailty in LSS is associated with increased symptom severity and greater disability related to LSS. In the present study, consistent with the findings of Kim et al. [7], disability was greater in patients with frail LSS and was associated with higher frailty levels. Neurogenic claudication and walking intolerance specific to LSS may also contribute to balance deterioration [1,27,28]. Muscle weakness, exhaustion, reduced mobility, and low physical activity associated with frailty, when superimposed on these existing symptoms, may lead to more severe impairments in physical functions in LSS and greater disability [7,9]. In the present study, handgrip strength was lower and pain intensity was greater in patients with frail LSS. Disability is also more pronounced in patients with frail LSS, as decreased handgrip strength [8] and increased pain intensity are associated with greater disability in LSS [20]. Given that the prevalence of both LSS and frailty increases in an ageing society, assessing frailty in patients with LSS is important for maintaining functional independence. In this way, the negative impact of frailty on disability management may be mitigated.

In LSS, spinal deformities and generalized muscle weakness are associated with an increased risk of falls [29]. When frailty is superimposed on LSS, fear of falling may increase due to the elevated risk of falls. This study demonstrated for the first time that patients with frail LSS have greater fear of falling than nonfrail patients. Additionally, higher frailty levels were moderately associated with greater fear of falling. Previous studies have shown that fear of falling is associated with an increased risk of falls in older frail adults [12,36,37]. Qin et al. [37] reported that the likelihood of frailty was 7.2 times higher in individuals with fear of falling. Therefore, fear of falling should be examined in relation to frailty [37]. Frailty may also exacerbate symptoms such as disability, muscle weakness, and physical inactivity observed in LSS [7,9], partly through its effects on neuromuscular function [9]. Therefore, patients may become more sensitive to falling during activities of daily living, leading to increased fear of falling. It has been reported that TUG test times ≥13.5 s indicate an increased risk of falls in older adults [38]. According to the TUG test, which is frequently used to assess mobility in LSS [21], patients with frail LSS in our study demonstrated a higher risk of falls than nonfrail patients, with test durations exceeding 13.5 s. These results suggest that frailty is associated with an increased risk of falls in patients with LSS, contributing to fear of falling and negatively affecting both psychosocial well-being and physical health. Additionally, fear of falling may restrict activities, thereby increasing vulnerability to frailty [12]. Thus, this bidirectional relationship between frailty and fear of falling warrants further examination to maintain health in patients with LSS.

Ageing negatively affects quality of life by reducing both mental and physical functions [39]. Symptoms related to LSS also worsen quality of life. Otani et al. [40] reported that LSS has a stronger adverse effect on quality of life than comorbid conditions such as osteoarthritis and respiratory, cardiovascular, hypertensive, and diabetic diseases. Frailty and quality of life are also known to have a bidirectional relationship [41]. The present study demonstrated poorer outcomes across all quality-of-life parameters, both physical and mental, in patients with LSS accompanied by frailty compared with those without frailty. As frailty levels increased in LSS, quality of life progressively deteriorated. Frailty diminishes functional capacity through reduced muscle strength, physical inactivity, and exhaustion [9]. This may result in decreased energy levels and increased fatigue in patients. This study demonstrated that a one-point decrease in vitality, a quality-of-life subscale in patients with LSS, corresponded to a 3.2% higher likelihood of frailty. Our findings provide additional evidence supporting a bidirectional relationship between frailty and quality of life in LSS. Frailty may further impair quality of life through its links to social isolation and depressive symptoms [41]. Therefore, the physical and psychological burdens associated with frailty contribute to declining quality of life in patients with LSS.

Considering these findings, rehabilitation programs should be individually tailored, as individuals with frail LSS experience greater pain and disability, along with poorer balance, increased fear of falling, and reduced quality of life. Early pain reduction is particularly important, given its relationship with impaired functional performance [3,7]. Additionally, balance-oriented rehabilitation may be effective in enhancing mobility and quality of life by reducing fear of falling and may also contribute to lowering frailty.

This study has several limitations. First, this was a cross-sectional study, which limits the ability to establish a cause-and-effect relationship between frailty and symptoms. Furthermore, there is conceptual overlap between the physical performance components included in the frailty assessment and outcome measures such as the SLST and TUG tests. This overlap may have influenced the strength of the observed relationships. Therefore, conceptual similarities among the measurement tools should be considered when interpreting the results. Additionally, because LSS severity was not recorded, its relationship with frailty could not be evaluated. The relationship between LSS severity and frailty may influence symptom severity and should be investigated in future studies. This limitation may also affect the generalizability of the findings from this single-center study. Patients’ static and dynamic balance was assessed using simple and practical tests frequently employed in clinical settings for LSS [20,21]. To better understand the mechanisms underlying the relationship between frailty and balance, future studies should incorporate objective assessment methods, such as stabilometric platforms, to provide more detailed information.

## 5. Conclusion

In conclusion, patients with frail LSS experience greater pain intensity, balance impairment, disability, fear of falling, and poorer quality of life than nonfrail patients. Furthermore, decreased mobility, physical activity, and vitality are associated with a higher likelihood of frailty in patients with LSS. Therefore, frailty substantially affects patients with LSS, both physically and psychosocially. Thus, early assessment of frailty in patients with LSS, along with the implementation of individualized rehabilitation programs, may represent an effective strategy to mitigate the negative impact of frailty on clinical outcomes.

## Figures and Tables

**Table 1:** Demographic characteristics of frail and nonfrail patients with LSS.

Variables	Frail (n=43) Mean±SD Median (IQR_25%–75%_)	Nonfrail (n=48) Mean±SD Median (IQR_25%–75%_)	p
Age (years)	65.91±7.68	64.15±6.96	0.254
Sex (n, %)			
Female	35 (81.4%)	31 (64.6%)	0.073
Male	8 (18.6%)	17 (34.4%)	
Height (cm)	161.88±7.23	164.33±7.99	0.131
Weight (kg)	80.16±8.46	81.93±11.25	0.402
BMI (kg/m^2^)	30.68±3.52	30.32±3.52	0.632
Hypertension (n, %)	25 (58.1%)	21 (43.8%)	0.170
Diabetes mellitus (n, %)	18 (41.9%)	14 (29.2%)	0.205
Cardiovascular disease (n, %)	5 (11.6%)	5 (10.4%)	0.854
Respiratory disease (n, %)	4 (9.3%)	2 (4.2%)	0.324
Duration of symptoms (month)	24 (24–36)	26 (15–57)	0.771
Affected side (n, %)			
Right	11 (25.6%)	18 (37.5%)	0.462
Left	17 (53.1%)	15 (31.3%)	
Bilateral	15 (34.9%)	15 (31.3%)	
Handgrip strength (kg)	18.30±4.86	27.35±7.91	**<0.001** *
4 m walk test (s)	10.05±2.70	6.86±2.59	**<0.001** *

BMI: body mass index.

* p < 0.05.

Bold font indicates statistically significant values.

**Table 2:** Frailty components in frail and nonfrail patients with LSS.

Variables	Frail (n=43)	Nonfrail (n=48)	p
Weight loss, n (%)	2 (4.7%)	4 (8.3%)	0.480
Muscle weakness, n (%)	33 (76.7%)	8 (16.7%)	**<0.001** *
Reduced mobility, n (%)	41 (95.3%)	17 (35.4%)	**<0.001** *
Low physical activity, n (%)	38 (88.4%)	16 (33.3%)	**<0.001** *
Exhaustion, n (%)	35 (81.4%)	8 (16.7%)	**<0.001** *

* p < 0.05.

Bold font indicates statistically significant values.

**Table 3:** Comparison of clinical characteristics between frail and nonfrail patients with LSS.

Variables	Frail (n=43) Mean±SD Median (IQR_25%–75%_)	Nonfrail (n=48) Mean±SD Median (IQR_25%–75%_)	p
NRS, low back pain	9 (8–9)	8 (5.25–9)	**<0.001** *
NRS, leg pain	9 (8–10)	8 (6–9)	**<0.001** *
SLST (s)	9.15±4.70	13.83±5.36	**<0.001** *
TUG (s)	13.85±3.51	9.95±2.40	**<0.001** *
ODI	59.16±18.20	43.16±15.68	**<0.001** *
FES-I	36.72±12.13	25.89±6.90	**<0.001** *
IPAQ total score (MET-min/week)	266 (138–478)	1583 (232.75–2209.75)	**<0.001** *
SF-36			
Physical functioning	29.30±20.60	48.22±28.64	**0.001** *
Physical role	0 (0–25)	25 (0–100)	**0.001** *
Bodily pain	32.26±22.87	53.43±19.19	**<0.001** *
Vitality	28.37±19.63	52.70±23.58	**<0.001** *
Social functioning	43.82±21.56	64.02±24.99	**<0.001** *
Emotional role	0 (0–33.33)	50 (0–100)	**0.002** *
Mental health	42.88±23.41	61.16±23.52	**<0.001** *
General health	29.09±16.21	45.20±20.47	**<0.001** *

NRS: numeric rating scale; SLST: single leg stance test; TUG: Timed Up and Go test; ODI: Oswestry Disability Index; FES-I: Falls Efficacy Scale–International; IPAQ: international physical activity questionnaire; SF-36: Short Form-36.

* p < 0.05.

Bold font indicates statistically significant values.

**Table 4:** Relationships between pain, balance, disability, physical activity, fear of falling, and quality of life and frailty.

Variables	Correlation coefficients (r)
NRS, low back pain	0.467**
NRS, leg pain	0.424**
SLST (s)	−0.538**
TUG (s)	0.626**
ODI	0.506**
FES-I	0.508**
IPAQ total score (MET-min/week)	−0.648**
SF-36	
Physical functioning	−0.450**
Physical role	−0.439**
Bodily pain	−0.496**
Vitality	−0.599**
Social functioning	−0.523**
Emotional role	−0.390**
Mental health	−0.493**
General health	−0.555**

NRS: numeric rating scale; SLST: single leg stance test; TUG: Timed Up and Go test; ODI: Oswestry Disability Index; FES-I: Falls Efficacy Scale–International; IPAQ: international physical activity questionnaire; SF-36: Short Form-36.

** p < 0.01.

**Table 5:** Logistic regression analysis of variables associated with frailty. (frail group, n = 43; nonfrail group, n = 48)

Independent variables	Frailty status		
	OR	95% CI	p-value
TUG (s)	1.482	1.183–1.856	**0.001** *
IPAQ total score (MET-min/week)	0.999	0.998–1.000	**0.007** *
SF-vitality	0.968	0.941–0.997	**0.031** *

TUG: Timed Up and Go test; IPAQ: international physical activity questionnaire; SF-36: Short Form-36, OR: odds ratio; CI: confidence interval.

* p < 0.05.

Bold font indicates statistically significant values.
